# Patient Transfer Decision Difficulty Scale: Development and psychometric testing of emergency department visits by long-term care residents

**DOI:** 10.1371/journal.pone.0210946

**Published:** 2019-02-01

**Authors:** Bor-An Chen, Hui-Hui Chien, Chun-Chung Chen, Hui-Tsai Chen, Chii Jeng

**Affiliations:** 1 School of Nursing, Taipei Medical University, Taipei, Taiwan; 2 Emergency Department, Keelung Hospital, Ministry of Health and Welfare, Keelung, Taiwan; 3 Nursing Department, Yuanshan Branch, Taipei Veterans General Hospital, Ilan, Taiwan; Nord University, NORWAY

## Abstract

**Background and objectives:**

Nurses serve as gatekeepers of the health of long-term care facility (LTCF) residents and are key members deciding whether residents should visit an emergency department (ED). Inappropriate decisions as to ED visits may result in ED overcrowding, excessive medical expenses, and nosocomial infections. Currently, there is a lack of effective tools for assessing the barriers and level of difficulty experienced by LTCF nurses. The purposes of this study were to develop a Patient Transfer Decision Difficulty Scale (PTDDS) and test its effectiveness.

**Methods:**

This study randomly sampled LTCFs in Taiwan and surveyed two or three nurses in every institution selected. Registered return envelopes were provided for participants to return self-completed questionnaires. Three steps were used to develop the scale and items: in step I, the instrument was developed; in step II, psychometric testing was conducted, which entailed performing an exploratory factor analysis (EFA) to verify the construct validity and reliability of the developed items; and in step III, a confirmation study was conducted using a confirmatory factor analysis (CFA) and structural equation modeling to cross-validate the factors and items.

**Results:**

The cumulative sum of variance explained by the measurement models of the three factors in the PTDDS was 63.54%.When deciding whether to transfer LTCF residents to EDs, the most pronounced barrier experienced by nurses were for judging the severity of “clinical episodes”, which had an explanatory power of 37.49%. The second and third pronounced barriers and decision difficulty experienced by nurses were “communication and information” and “timing of the residents’ emergency visits,” which explained 16.81% and 9.24% of the variance, respectively.

**Conclusions:**

The cross-validation results obtained using the EFA and CFA showed favorable reliability and validity of the PTDDS. For future studies, this study recommends performing large-scale investigations of the level of decision difficulty and related factors experienced by nurses in LTCFs of varying levels and types.

## Introduction

It is estimated that between 2010 and 2050, the global elderly population will increase 2.86-fold. Following currents trends of global population aging and an increasing average life expectancy [1], the number of long-term care facility (LTCF) residents has increased. In Taiwan, most LTCF s do not equip with doctors for 24 hours. Doctors only visit facilities at regular time and cannot give consultation to LTCF nurses at all times. Because no regulations are currently in place stipulating that resident physicians be available in LTCFs [2,3], and since LTCF residents are generally older adults with multiple comorbidities such as fever, urinary tract infections, asthma, unstable blood pressure, altered state of consciousness, and unexpected accidents [4–7], the number of LTCF residents being transferred to emergency departments (EDs) because of unexpected acute medical needs is substantially higher than those in other age groups [5,6,8]. Nurses serve as gatekeepers of LTCF residents’ health [9] as well as key decision makers for whether residents visit EDs [2,10].

Many factors influence decisions concerning whether to transfer an LTCF resident to the ED to treat an unexpected acute medical need. These factors vary considerably in characteristics and include the severity of the resident’s health problems [7,11], whether the resident has signed a consent form for advance care planning and an advance directive [12,13], residents and their family members’ wishes [6,9], whether LTCF systems and resources are sufficient [14], resident families’ economic status [5,15], and nurses’ confidence and professional competence [16]. Because of the numerous factors that must be considered, such as the lack of standard operating procedures, and patients' or their family members’ uncertain or ambiguous contextual factors, when making decisions regarding a resident’s ED visit, nurses experience distress, barriers, stress, and anxiety and may make improper decisions [16–18]. Studies showed that most LTCF nurses’ decisions to transfer LTCF residents to EDs were inappropriate [2,7]. For example, in one study, approximately 1/4 of residents had visited an ED in the previous 6 months [10], but only 35.30% of them were classified as emergent triage [10]. In other studies, 13%~50% of residents did not need to visit the ED, and 19%~67% of them did not need to be hospitalized [5,14,19]. Unnecessary ED visits or misuse of ED resources may result in ED overcrowding [20], higher nosocomial infections, and emergency medical care-related iatrogenic adverse effects [21], increases in burdens of medical expenses, decreases in family members’ satisfaction levels [21,22], and increased nurse turnover rates because of anxiety, frustration, and a lack of a sense of accomplishment [23]. These all seriously influence the quality of long-term care delivery systems.

Moreover, relevant studies showed that by making proper medical decisions, nurses can reduce residents’ hospitalization and ED visits by 30% and 54.1%, respectively [24–26]. Because of the shortage of effective tools for assessing the barriers and level of difficulty experienced by LTCF nurses when deciding whether to transfer an LTCF resident to the ED, the purposes of this study were to develop a Patient Transfer Decision Difficulty Scale (PTDDS) and test its effectiveness.

## Methods

### Study design

A cross-sectional survey was conducted in this study. According to our previous qualitative study results, the PTDDS was preliminarily developed. Subsequently, a psychometric test was performed, and structural equation modeling (SEM) was adopted to cross-validate the effectiveness of the PTDDS.

### Sample and setting

Registered nurses who work as full-time nurses of LTCF were included. Nurses who had participated in the earlier qualitative research were excluded. Participants were recruited as follow procedures. At first, a list of LTCFs was obtained from the Taiwanese Ministry of Health and Welfare website [27]. A random number table generated by a computer was used to randomly sample LTCFs. The authors called a supervisor or director of each LTCF to explain the study background and motivation, and ask if they were willing to participate in this study. For every LTCF that declined the invitation, an additional LTCF was selected from the LTCF waiting list. Then, the supervisor or director of each LTCF which were willing to participate in this study selected 2–3 nurses who met the selection criteria to answer a Patient Transfer Decision Difficulty Scale (PTDDS).

First, 338 facilities were selected by simple random sampling from the whole country. Through phone calls, 53 facilities refused to participate in the research because the supervisor or person in charge has no intention or the facility was too busy handling Institutional evaluation. However, Among the 53 facilities refused to participate in the research, there were 20 in the northern part, 10 in the central, 18 in the southern, and 5 in the eastern part of Taiwan. While among the 285 facilities agree to participate, there were 103 in the north, 63 in the central, 100 in the south, and 20 in the east. The proportion of representatives between facilities which refused to participate in the research and facilities which participate in are similar. Then, according to the scale of beds of facilities, 2 copies of questionnaires were sent to 213 facilities and 3 copies were sent to 72 facilities respectively. Participants were required to self-complete the questionnaires and mail them back using the provided return envelopes within 2 weeks. The response rate was 86.71%, recovering 556 questionnaires among 642 copies sent. Eighty-six questionnaires were not responded because the subject was too busy to fill in, missing of the questionnaire or missing during reply. The authors checked every questionnaire to determine whether it was completed in full, and deleted those with more than 50% missing data as well as those exhibiting a floor or ceiling effect [28].

### Study procedures

**Fig 1:**
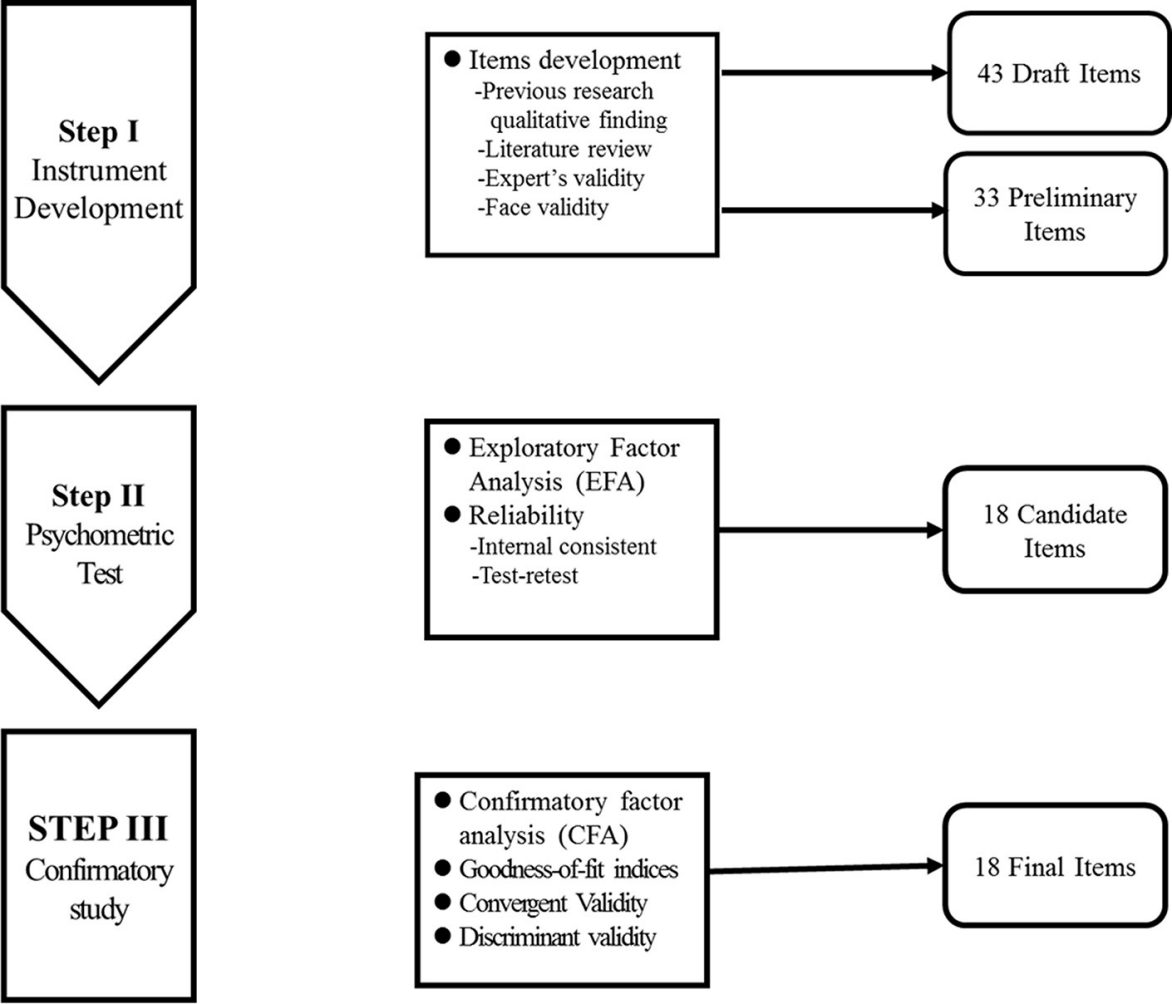
Steps for development and validation of PTDDS.

Ethics approval for the study was granted by the Taipei Medical University Joint Institution Review Board (TMU-JIRB: N201704040). After participants had signed the informed consent, the questionnaires were anonymously collected. The study procedure was divided into three steps (Fig 1).

### Step1: Item development

After referencing qualitative results obtained in our previous study “LTCF Nurses’ Experience of Transferring LTCF Residents to Emergency Departments”, we also conducted comprehensively literature search in electronic resources of PubMed added CINAHL, Scopus, and Google Scholar for auxiliary resources between 2000 and 2017, which were limited to English language and full text of scientific articles. In the search strategy, which utilized utilization of “long term care facility” or “nursing home” was title word terms for identifying the scope of searching literatures. In the searched scope, we used Medical Subject Heading (MeSH) terms or free term “transfer” or “transition” or “referral” or “Emergency” combined with “readmission” or “hospitalization”, which were an iterative process to obtained article and have searched manually the bibliographic references of selected article. Finally, 43 draft items in total were selected for the PTDDS.

To enhance the questionnaire’s importance, applicability, representativeness, and clarity, four professors who specialize in the field of long-term care and three LTCF nurses with master’s degrees performed two rounds of expert validity reviews of the draft items. Items with an item-level content validity index of <0.78 were deleted [29], and an overall scale-level content validity index of ≥0.8 was considered to indicate favorable content validity [30]. Subsequently, to conduct the Face Validity, the authors invited one acquaintance who met the inclusion criteria from each facility in the north, central, south, and east of Taiwan. In a way of snowballing, the authors obtained 6 subjects in the north, 5 in the central, 6 in the south, and 3 in the east, totaled 20 subjects. Through face to face interview, inappropriate items were revised or deleted. Draft items that passed the expert and face validity tests were selected as the preliminary items.

### Step 2: Psychometric test

A five-point Likert scale was adopted to measure the preliminary items, where scores of 0, 1, 2, 3, and 4 respectively signified never, rarely, sometimes, often, and always experiencing decision-making difficulty. This study conducted an exploratory factor analysis (EFA) on data of 200 participants to analyze the construct validity of the items, in which those with favorable and unfavorable construct validity were respectively retained and deleted, thereby producing the candidate items. Next, the reliability of each candidate item was tested.

#### EFA

Preliminary items were analyzed using the SPSS 22 software package (SPSS IBM, New York, USA). The Kolmogorov-Smirnov test was used to test if the data are normal distribution (p>.05). Prior to conducting the study, a Kaiser-Meyer-Olkin (KMO) test to measure the sampling adequacy (with a cutoff of >0.50) and Bartlett’s test were performed to verify whether significant differences existed between the data, to determine whether the data could undergo an EFA [31], and identify communality between test items. A maximum likelihood (ML) estimation was employed to extract factors, for which factors with an eigenvalue of >1 were selected, and factors between items were obtained using the oblique minimum rotation method. EFA and scree tests were repeatedly performed, and the following criteria were used to determine items to be retained and the appropriate number of factors to be utilized: a factor loading of ≥0.60 [32], no cross-loading factors between items [33], and a factor with at least three items [31]. Therefore, the construct validity of the PTDDS was established.

#### Reliability

To ensure the internal consistency of the PTDDS, the following conditions were used to determine items to be deleted: a Cronbach’s α of <0.70 [34] and a corrected item to total correlation of <0.30 [35]. According to the sequence of recovering questionnaires, the authors picked the first 50 subjects to perform two-week retest of reliability. In addition to the intraclass correlation coefficient (ICC) was computed, Pearson correlation coefficient (with a cutoff of ≥0.70) [35] and a paired *t*-test (with a cutoff of *p*>0.05) were used to verify the stability of the PTDDS [36].

### Step 3: Confirmation study

#### Confirmatory factor analysis (CFA)

To verify the accuracy of the EFA results, CFA and SEM analysis were performed on data of the other 348 participants, in which relationships between the item and factor measured models were established. Subsequently, an analysis was conducted using SPSS/AMOS vers. 22.0 (SPSS IBM), and estimates were made using the maximum likelihood estimation (MLE) method. Prior to the analysis, the measurement models were evaluated to determine whether they were suitable for calculating the estimates of the SEM framework [37]. Next, the goodness of fit, convergent validity, and discriminant validity of the measurement models as well as the latent construct relationships between candidate items were assessed. Then, cross-validation was performed to determine the construct validity of the EFA [33,38].

#### Goodness-of-fit indices (GFIs)

Several methods exist for testing GFIs of an SEM framework [39]. In this study, the absolute fit method was adopted, which featured the following parameters: Chi-square (*p*>0.05), *X*^*2*^*/Df* (with a cutoff of ≤3) [40], and root mean square error approximation (RMSEA) (0.05<cutoff<0.08, good fitting) [41]. The cutoff value of ≥0.90 of the model fit indices, including the Goodness-of-fit index (GFI), adjusted GFI (AGFI), comparative fit index (CFI), Tucker-Lewis index (TLI), and normed-fit index (NFI), was considered suitable [41].

#### Convergent validity

The convergent validity of the SEM had to satisfy the following conditions: (1) the items had to show a standardized factor loading (λ) of ≥0.50 and a *t*-value that reached a level of significance (*p*≤0.05) [33]; (2) latent variables had to show a construct reliability (CR) of ≥0.70 [37,42]; and (3) the latent variables had to show an average variance extracted (AVE) of ≥0.50 [37,42].

#### Discriminant validity

A discriminant validity analysis was conducted to verify whether the latent variables belonged to different concepts to avoid excessive overlapping of their meanings [33]. The discriminant validity for the SEM framework had to satisfy the following condition: the square root of AVE of each latent variable (factor) had to be greater than the correlation coefficients between the latent variables [37].

## Results

### Sample characteristics

In total, 556 questionnaires were received from participants for a response rate of 86.71%. Because eight of the questionnaires were invalid (three having more than 50% missing data and five exhibiting a ceiling or floor effect), they were excluded from the statistical analysis; thus, only 548 questionnaires were used. Study participants were mostly female (97.45%), mostly married (61.5%), mostly college graduates or above (92.2%), and had an average age of 39.10±0.49 years. Furthermore, they had 12.95±0.42 years of nursing experience and 5.76±0.23 years of LTCF nursing experience. Other demographic information and nursing backgrounds of participants are shown in Table 1.

**Table 1:** Demographic and nursing characteristics (*N* = 548).

Demographics	
Age, years; mean (SD)	39.10 (10.31)
Gender (female, %)	97.45
Education	
Senior high school (%)	7.8
Junior college (%)	46.9
University (%)	41.2
Graduate institute (%)	4.1
Marital status	
Single (%) (divorced, widowed, or in a relationship without living together)	38.5
Married (%)	61.5
Nursing-related characteristics	
Number of years working as nursing personnel, mean (SD)	12.95 (0.42)
Number of years working as LTCF nursing personnel, mean (SD)	5.76 (0.23)

Note: LTCF, long-term care facility; SD, standard deviation.

### Step 1: Instrument development

#### Item generation and reduction

After reviewing the literature and referencing previously obtained qualitative study results, this study generated 43 draft items for the PTDDS. Two rounds of expert validity reviews were performed on the draft items, during which items with an item-level content validity index of <0.78 (i.e., Q1, Q2, Q3, Q4, Q10, Q18, Q19, Q21, Q27, and Q33; a total of 10 items) were deleted. Subsequently, interviews were conducted with 20 participants to test the face validity of the items, and participants indicated that the wording and format of the items were appropriate. Therefore, the remaining 33 items were retained to form the preliminary items.

### Step 2: Psychometric test

#### EFA results

A KMO test for measuring sampling adequacy and Bartlett’s test of sphericity were performed on the 33 preliminary items; all items showed a KMO value of ≥0.80, and Bartlett’s test result reached a significant level (*p*<0.001). The result of Kolmogorov-Smirnov test indicated normal distribution of the data (p = 0.2). The overall mean value (standard deviation) of subjects = 44.27(20.42). The median was 44 and Skewness = 0.181±0.172, which all indicated there was no problem with floor and ceiling effects and Skewness.

From four EFAs combined with scree tests, three common factors were extracted. Items with a factor loading of <0.60 (i.e., Q14, Q15, Q16, Q17, Q20, Q28, Q29, Q30, Q32, Q34, and Q35; a total of 11 items) were deleted. In addition, because Q36, Q37, Q38, and Q40 had a factor loading of ≥0.60 in both factors 2 and 3, they were cross-loaded and thus were deleted. For this step, 15 items were deleted, leaving 18 candidate items. Factor 1 contained eight items, featured an eigenvalue of 6.75, and explained 37.49% of the variance. Factor 2 contained six items, featured an eigenvalue of 3.03, and explained 16.81% of the variance. Factor 3 contained four items, featured an eigenvalue of 1.66, and explained 9.24% of the variance. The cumulative sum of variance explained by the three factors was 63.54% (Table 2).

**Table 2:** Results of maximum likelihood (*N* = 200).

No.	Item	Factor1	Factor2	Factor3
7	Observed abnormal pulse rhythm	0.848		
8	Observed abnormal breathing	0.836		
11	Observed hematuria	0.770		
12	Observed coffee-ground emesis	0.752		
9	Observed fluctuating abnormal blood pressure	0.712		
5	Resident experienced changes in consciousness status	0.705		
13	Resident reported 4 points or more on the pain rating scale	0.694		
6	Abnormal body temperature occurred twice within 1 week	0.665		
25	Interaction between a resident and his or her family members		0.833	
22	Trusting relationship between nurses and resident’s family members		0.809	
26	Signing of a “do not resuscitate” (DNR) consent form		0.731	
31	Availability of oxygen supply equipment		0.716	
23	Resident’s willingness to visit an emergency department for medical care		0.687	
24	Family members’ willingness for the resident to visit an emergency department for medical care		0.663	
42	The frequency of resident being transferred to an emergency department influenced the LTCF’s reputation			0.864
41	Emergency department staff doubted the timing of the resident’s visit to an emergency department for medical care			0.705
43	The frequency of a resident being transferred to an emergency department influenced the LTCF’s revenue			0.680
39	Possible Medical disputes			0.640
Eigenvalue		6.75	3.03	1.66
Explained sum of the variance (%)		37.49	16.81	9.24
Cumulative explained sum of the variance (%)		37.49	54.30	63.54

### Reliability

The internal consistency of the 18 candidate items was analyzed using Cronbach’s α; factors 1, 2, and 3 showed Cronbach’s α values of 0.91, 0.88, and 0.81, respectively, and Cronbach’s α for of the entire PTDDS was 0.90. The corrected item to total correlation coefficients of the 18 items all exceeded 0.30 (ranging 0.426~0.632). Characteristics of the internal consistency reliability of the 18 items are shown in Table 3. According to questionnaires sent to 50 participants and the test-retest performed 2 weeks apart, the ICC was 0.909 (CI: 0.846–0.948). The ICC of three subscales including “Clinical episodes”,” Communication”, and “Timing” were 0.857 (CI:0.761–0.916), 0.862 (CI:0.769–0.919), and 0.840 (CI:0.734–0.906) respectively. The Pearson correlation coefficient of the 18 candidate items equaled 0.87, and no significant differences existed between the two test results (*t* = 1.781, *p* = 0.081).

**Table 3:** 18 Item characteristics of internal consistency (*N* = 200).

No.	Item	Cronbach’s α (if item deleted)	Corrected item-total correlation
5	Resident experienced changes in consciousness status	0.891	0.522
6	Abnormal body temperature occurred twice within 1 week	0.889	0.587
7	Observed abnormal pulse rhythm	0.888	0.626
8	Observed abnormal breathing	0.888	0.621
9	Observed fluctuating abnormal blood pressure	0.890	0.554
11	Observed hematuria	0.888	0.632
12	Observed coffee-ground emesis	0.888	0.622
13	Resident reported 4 points or more on the pain rating scale	0.890	0.559
22	Trusting relationship between nurses and resident’s family members	0.893	0.473
23	Resident’s willingness to visit an emergency department for medical care	0.892	0.490
24	Family members’ willingness for the resident to visit an emergency department for medical care	0.889	0.569
25	Interaction between a resident and his or her family members	0.893	0.460
26	Signing of a “do not resuscitate” (DNR) consent Form	0.890	0.559
31	Availability of oxygen supply equipment	0.894	0.451
39	Possible Medical disputes	0.894	0.426
41	Emergency department staff doubted the timing of the resident’s visit to an emergency department for medical care	0.893	0.441
42	The frequency of resident being transferred to an emergency department influenced the LTCF’s Reputation	0.888	0.621
43	The frequency of resident being transferred to an emergency department influenced the LTCF’s Revenue	0.890	0.557

### Step 3: Confirmatory study

#### CFA results

According to the PTDDS, the variance in the standard errors of all items ranged 0.153~0.897 (i.e., >0); all *t-*values reached a level of significance (*p*<0.05); no abnormal standardized regression weights (λ) (i.e., λ>1) were observed for any of the items (all λ values ranged 0.630~0.924); and standard errors were not too large (i.e., within the range of 0.027~0.076), indicating that the measurement models did not possess offending estimates and were suitable for the goodness-of-fit test in SEM (Table 4).

**Table 4:** Results of the confirmation study (*N* = 348).

Latent variable	Item	λ	Error variable			Convergent validity			Discriminant validity	
			Variance	S.E.	*t-*value	CR	AVE	λ^2^	√_AVE_	Correlation
Clinical episode						0.912	0.568		0.754	0.327^a^
	Q7	0.807	0.299	0.027	11.063***			0.651		
	Q8	0.842	0.312	0.030	10.433***			0.709		
	Q11	0.788	0.330	0.029	11.328***			0.621		
	Q12	0.760	0.412	0.035	11.639***			0.578		
	Q9	0.785	0.377	0.033	11.364***			0.616		
	Q5	0.656	0.628	0.051	12.326***			0.430		
	Q13	0.630	0.539	0.043	12.438***			0.397		
	Q6	0.736	0.448	0.038	11.848***			0.542		
Communication and information						0.898	0.595		0.771	0.502^b^
	Q25	0.838	0.380	0.039	9.844***			0.702		
	Q22	0.807	0.553	0.052	10.529***			0.651		
	Q31	0.709	0.897	0.076	11.761***			0.503		
	Q26	0.716	0.672	0.057	11.696***			0.513		
	Q23	0.804	0.462	0.044	10.584***			0.646		
	Q24	0.744	0.538	0.047	11.431***			0.554		
Timing						0.868	0.625		0.790	0.369^c^
	Q41	0.796	0.392	0.038	10.411***			0.634		
	Q42	0.924	0.153	0.030	5.108***			0.854		
	Q39	0.702	0.594	0.051	11.750***			0.493		
	Q43	0.720	0.552	0.048	11.577***			0.518		

* *p*<0.05

** *p*<0.01

*** *p*<0.001.

λ, standardized regression weight (factor loading); AVE, average variance extracted; CR: construct reliability; √_AVE_: square root of the AVE

a, Clinical episode-Communication and information factor correlation coefficient

b, Clinical episode-Timing factor correlation coefficient

c, Communication and information-Timing factor correlation coefficient.

#### GFIs

Among the GFIs in SEM for verifying the measurement models, except for the Chi-squared test, the results of which showed significant differences (*p*<0.001) due to the large sample size and thus did not meet the standard, all indices met the standards, namely AGFI = 0.874, which was close to 0.90; GFI, CFI, TLI, and NFI were all ≥0.90; RMSEA = 0.067, which was slightly lower than the standard value of 0.80; and the X^2^/df ratio was 2.557 which was <3, which denoted favorable fitness (Table 5).

**Table 5:** The model goodness-of-fit.

Index	Cutoff	Evaluation	
		Value	Rank
Absolute fit			
χ2	*p*>0.05	337.567***	-
*Df*	-	132	-
χ^2^/*df*	≤3	2.557	Good fit
GFI	≥0.90	0.902	Good fit
AGFI	≥0.90	0.874	Acceptable
SRMR	≤0.08	0.058	Good fit
RMSEA	≤0.08	0.067	Good fit
Incremental fit			
NFI	≥0.90	0.911	Good fit
TLI	≥0.90	0.934	Good fit
CFI	≥0.90	0.943	Good fit

* *p*<0.05

** *p*<0.01

*** *p*<0.001.

χ2, Chi-squared; *Df*, degrees of freedom; GFI, goodness-of-fit index; AGFI, adjusted GFI; SRMR, Standardized Root Mean Squared Residual; RMSEA, root mean squared error approximation; NFI, normed-fit index; TLI, Tucker-Lewis index; CFI, comparative fit index.

#### Convergent validity

The purpose of assessing the convergent validity is to verify whether multiple items included in a latent variable are aggregated in a latent variable. For the standardized factor loading (λ) of the 18 items in the SEM framework, only Q5 (λ = 0.656) and Q13 (λ = 0.630) were slightly lower than the standard cutoff of 0.70; all of the remaining 16 items featured a standardized factor loading of ≥0.7. The CR of the latent variables of factors 1~3 were 0.912, 0.898, and 0.868, respectively, satisfying the ≥0.70 requirement. In addition, the AVEs of the latent variables all exceeded 0.50 (ranging 0.568~0.625), showing that the various items in the PTDDS and the latent variables to which they belonged demonstrated favorable convergent validity (Table 4).

#### Discriminant validity

Square roots of the AVEs of the three latent variables were all greater than the correlation coefficients between the latent variables, indicating that the items and latent variables of the SEM framework exhibited favorable discriminant validity, and that the latent variables (dimensions) entailed different concepts (Table 4).

**Fig 2:**
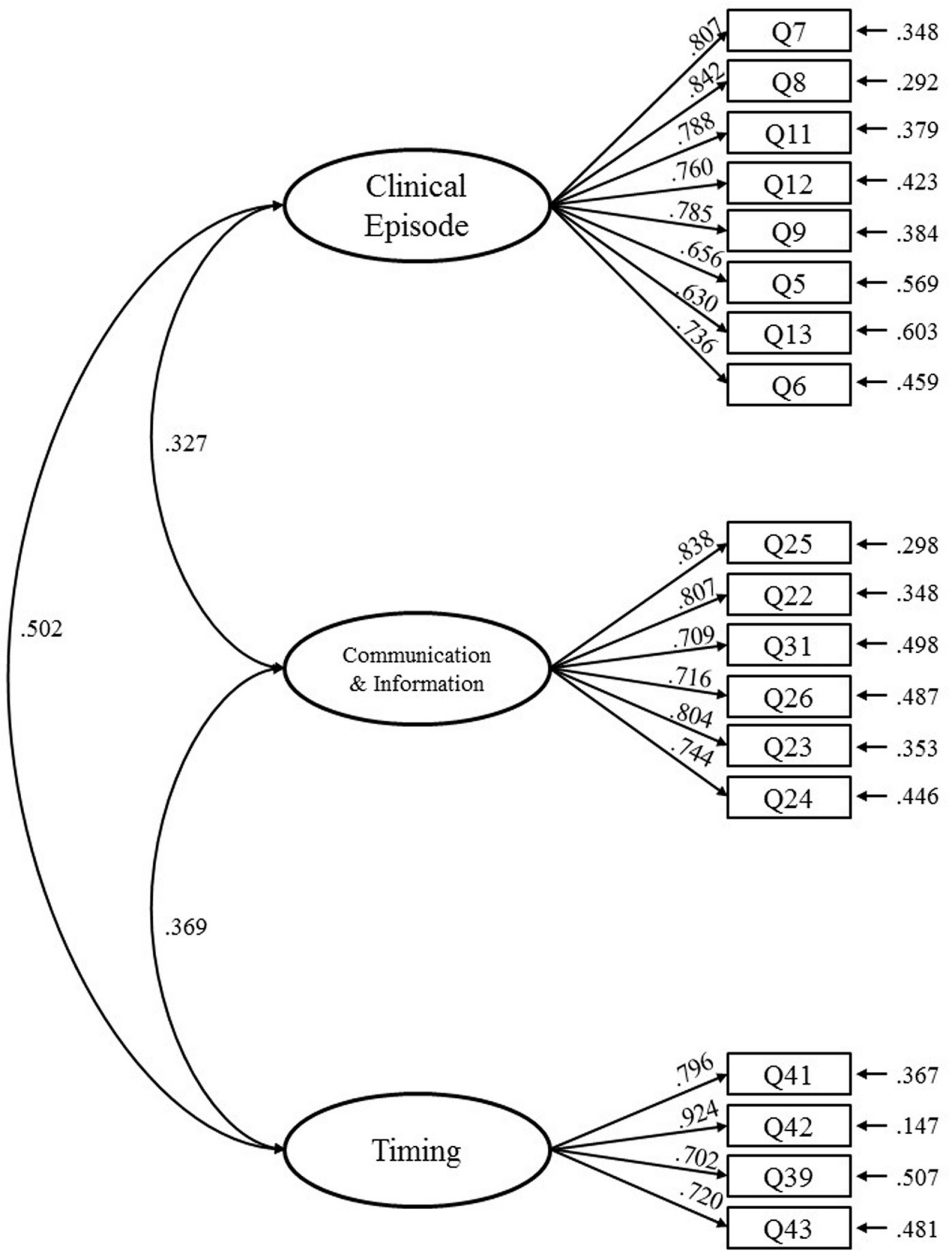
The PTDDS measurement model of confirmatory factor analysis.

The PTDDS measurement model did not have offending estimates and displayed favorable GFIs, convergent validity, and discriminant validity, indicating that it possessed favorable internal and external SEM quality (Fig 2).

## Discussion

The objective of this study was to develop a favorable psychometric tool that can be used to assess barriers and level of difficulty experienced by LTCF nurses when making decisions regarding emergency visits by LTCF residents. Study results showed that the PTDDS possessed Cronbach’s α of >0.70, a corrected item to total correlation coefficient of >0.30, and non-significant differences between test-retest results held 2 weeks apart. These results showed that the PTDDS featured favorable internal consistency, reliability, and retest stability. A CFA was used to verify the EFA, where the measurement models constructed using the SEM supported the three factor models. Most GFIs exceeded cutoff values and suggested favorable goodness-of-fit, convergent, and discriminant validities. The SEM framework exhibited favorable internal and external quality, and the study results confirmed that the PTDDS possessed excellent reliability and validity.

Results of the factor analysis showed that the PTDDS could be divided into the following dimensions that reflect barriers and level of difficulty experienced by LTCF nurses when deciding whether to transfer LTCF residents to EDs: (1) clinical episodes; (2) communication and information; and (3) timing of the residents’ emergency visits.

The clinical episodes dimension denotes that LTCF residents have fluctuating physical conditions and multiple comorbidities, making it difficult for LTCF nurses to assess their health condition and causing nurses to face dilemmas and experience distress when deciding whether to transfer residents. Studies showed that LTCF residents’ unexpected ED visits are generally caused by clinical emergencies such as sudden changes in their physiological signs, changes in their mental states, and accidents [43]; nurses’ care experience, judgment, and ability to handle these situations are the main factors determining whether a resident is transferred to an ED when a clinical episode occurs [2,44]. Other studies showed that improving LTCF nurses’ ability to accurately assess and handle LTCF’s residents’ health situations can decrease the occurrence of inappropriate ED visits [25,26,45], thereby lowering nurses’ distress levels. This study found that the clinical episodes dimension had an explanatory variance of 37.49%, revealing that when deciding whether to transfer an LTCF resident to an ED, the most pronounced barrier or difficulty experienced by nurses was judging the severity of a clinical episode.

The second most pronounced factor influencing the difficulty experienced by nurses was “communication and information”, which denotes environmental and situational factors influencing LTCF residents’ visits to EDs, when LTCF nurses find that the a resident’s health status has deteriorated or when an accident has occurred. These factors include relationships and interactions between nurses and residents (and their family members), nurses possessing complete knowledge of a resident’s health information and communicating it with the residents’ family members, nurses understanding a resident’s and their family members’ willingness to and approval or disapproval of an ED visit by the resident, and nurses providing the resident with pre-hospital care-related treatment. The literature shows that LTCF nurses must consider residents’ individual needs and accordingly communicate and coordinate with relevant medical personnel to facilitate the provision of integrated and continuous care [46]. Therefore, LTCF nurses act as coordinators to maintain favorable interactive relationships with residents and their family members [8,7,11], thereby achieving effective communication. In addition, LTCF residents being at the end of their lives is also a reason for the need for unexpected emergency medical treatment. If LTCF nurses can communicate with end-of-life LTCF residents and their family members and provide them with hospice information and set up mutual advance care planning, the frequency and number of visits to EDs by these residents will decrease [13]. In clinical practice, researchers often find that communication and information sharing between LTCF nurses and family members are poor, causing inconsistencies in family members' willingness to approve emergency medical treatment, especially for senior citizens, those who are bedridden, and those with complex relationships between residents and family members.

In addition, this study found that “Q31: availability of oxygen supply equipment” was one of the variables in the communication and information dimension (λ^2^ = 0.502), which may be because oxygen use is a care treatment intervention that LTCFs can provide without a medical prescription before residents are transferred to an ED. Accordingly, notifying the residents’ family members whether oxygen treatment has been administered may be crucial to facilitating favorable nurse-resident and family member communication.

The timing of the residents’ emergency visits denotes the timeliness of LTCF residents’ emergency visits and relevant influencing factors. This is the third factor influencing LTCF nurses’ barriers and level of difficulty related to decision making as to residents’ ED visits. This finding is similar to results of our previous study, which asserted that timeliness is the most crucial factor for ED visits; nursing home healthcare providers must promptly identify health situations of residents and assess whether they are to be transferred to an ED [7]. Jablonski, Utz, Steeves, and Gray [47] found that nursing home residents’ family members may refuse to have a resident transferred to an ED in a timely manner to ensure that they retain their nursing home bed and prevent economic burdens from hospitalization and associated medical expenses. This problem is particularly pronounced if residents are older adults with significant declines in physical and mental functions and repeatedly visit the ED. This delay in an ED visit can subsequently result in a deterioration of a resident’s health and ED personnel questioning the nurses’ judgment in terms of the timeliness of the transfer. Although LTCF nurses wish to lower the economic burdens of residents’ family members and keep the resident in the LTCF to increase the LTCF's revenue, they also worry about the potential medical disputes that may arise by not sending a resident to the ED. Thus, they experience a psychological paradox. This finding was identical to qualitative study results obtained in the study “LTCF Nurses’ Experience of Transferring LTCF Residents to Emergency Departments” by the present authors, and constitutes the third factor of barriers and level of difficulty experienced by LTCF nurses.

The PTDDS explained only 64% of variance. The possible reasons that 36% of variance could not be explained might include the status of the resident, such as age, insufficient physiological monitoring equipment, and incomplete medical support system, e.g. there is no residential doctors for consultancy. Though LTCFs are equipped with emergency medication, airway management, and intubation equipment, nurses cannot use them independently due to regulations.

The facilities in this study were randomly sampled from across the country instead of local regions. The distribution ratio of the facilities among the north, central, south and east across the country in this study was similar to that of the data from the Ministry of Health and Welfare in Taiwan. According to the website of Ministry of Health and Welfare [48], gender ratio in the demographic data of nurses served in LTCFs, female accounts for 98.6% which is similar to 97.45% in this study.

### Limitations

The PTDDS was developed using a rigorous statistical analytical procedure. Although items that failed to meet the standards were deleted, those that were deleted because they were cross-loaded, (not because they had a factor loading of <0.60, e.g., Q36, Q37, Q38, and Q40) were still of value and important and merit attention in clinical practice. Because this study adopted a random questionnaire survey method and the questionnaires were delivered and retrieved through the mail, whether the survey results were influenced by the environment the participants were in, their mental state, or their personal history could not be determined. In addition, participants were assigned by the LTCF director, which may result in the potential source of sample distortion bias because of expectation and pressure of the director. In the future studies, using an E-Questionnaire to reduce the bias were suggested.

### Implications

The PTDDS can be used to assess difficulties experienced by LTCF nurses when deciding whether to transfer an LTCF resident to the ED as well as the reasons behind such decisions. In addition, the PTDDS can serve as a reference when formulating standard operating procedures for transferring LTCF residents to EDs, designing courses to strengthen the abilities of LTCF nurses and relevant personnel to communicate, adapt, and handle related situations, and enabling EDs to construct tele-medicare platforms to transfer LTCF residents to EDs in an effective, accurate, and timely manner, thereby enhancing the “bridging of care” between LTCFs and EDs. In the future, the PTDDS could be used to perform large scale stratified random sampling research to compare the difference in decision making in sending residents to emergency between different regions and types of facilities, and these results can be an important reference for government’s policy making.

## Conclusions

This study developed and tested the first ever tool for assessing barriers and level of difficulty experienced by LTCF nurses when deciding whether to send an LTCF resident to the ED. The PTDDS was found to possess the following advantages: (1) it was constructed using data obtained from LTCF nurses recruited randomly throughout Taiwan, and thus the sample displayed outstanding representativeness; and (2) it was developed through a multistep, rigorous development process and cross-validated using an EFA and CFA, enabling it to display favorable reliability and validity. Large-scale measurements may be made in the future to explore levels of difficulty experienced by nurses in LTCFs of varying levels and types when deciding whether to send an LTCF resident to the ED as well as related factors. We hope to improve the quality of decisions made by LTCF nurses concerning the transfer of residents to EDs, thereby enhancing LTCF care quality.

## Supporting information

S1 File (SAV)
